# Supplementary material for: Prevalence of unculturable bacteria in the periapical abscess: A systematic review and meta-analysis
Source: PLoS One. 2021 Aug 5;16(8):e0255485. doi: 10.1371/journal.pone.0255485 (PMC8341601; doi:10.1371/journal.pone.0255485)
Supplement: S1 Table — (PDF) [file pone.0255485.s002.pdf]

Source	Prevalence of uncultivable bacteria in dentoalveolar lesions
**PubMed**	Key words
**Search #1**	Periapical lesion* OR periapical granuloma* OR chronic periapical lesion* OR chronic periapical granuloma* OR apical granuloma* OR radicular granuloma* OR periapical cyst* OR dental lesion* OR chronic periapical cyst* OR apical cyst* OR radicular cyst* OR dental cyst* OR dentoalveolar lesion* OR periapical abscess* OR dentoalveolar abscess* OR dental abscess* OR dental granuloma* OR dentoalveolar cyst* OR dentoalveolar granuloma* OR radicular lesion* OR radicular abscess* OR apical lesion* OR apical abscess* OR acute dentoalveolar abscess* OR acute periapical abscess*
**Search #2**	Microorganism* OR microbiota OR microbiome* OR anaerobe* OR aerobe* OR pathogen* OR germs OR microbe* OR facultative anaerobe* OR microaerophile* OR micro-organism* OR bacteri*
**Search #3**	Uncultivable OR yet-to-be-cultivated OR unculturable OR uncultured OR uncultivated OR non-culturable OR non-cultivable OR non-cultivated OR non-cultured
**Search #4**	16S rRNA gene sequencing OR Temperature gradient gel electrophoresis TGGE OR Single-Strand Conformation Polymorphism SSCP OR Restriction fragment length polymorphism RFLP OR Terminal restriction fragment length polymorphism T-RFLP OR DNA microarray OR Fluorescence in situ hybridization FISH OR Quantitative polymerase chain reaction qPCR OR Sanger Sequencing OR first generation of sequencing OR next generation sequencing NGS OR Pyrosequencing (454 OR Illumina's Hi-Seq genome sequencers, OR Massively parallel signature sequencing OR Polony sequencing OR SOLiD sequencing OR Life Technologies SOLiD OR Ion semiconductor OR Ion Torrent sequencing OR Combinatorial probe anchor synthesis (cPAS-BGI/MGI OR Nanopore Sequencing OR DNA nanoball sequencing OR Heliscope single molecule sequencing OR Single molecule real time (SMRT sequencing OR DNA nanoball sequencing OR Heliscope single molecule sequencing OR Tunnelling currents DNA sequencing OR MALDI-TOF MS Mass spectrometry OR microfluidic Sanger sequencing OR Transmission electron microscopy DNA sequencing OR RNA polymerase RNAP sequencing OR Roche 454 OR Life Technologies Ion Torrent OR Pacific Biosciences OR third generation sequencing OR polymerase chain reaction PCR OR denaturing gradient gel electrophoresis DGGE OR real time polymerase chain reaction RTPCR
**Search #5**	#1 AND #2 AND #3
**Search #6**	#1 AND #2 AND #4
**Search #7**	#5 OR #6

Scopus

**Search #1**	periapical AND lesion* OR periapical AND granuloma* OR chronic AND periapical AND lesion* OR chronic AND periapical AND granuloma* OR apical AND granuloma* OR radicular AND granuloma* OR periapical AND cyst* OR dental AND lesion* OR chronic AND periapical AND cyst* OR apical AND cyst* OR radicular AND cyst* OR dental AND cyst* OR dentoalveolar AND lesion* OR periapical AND abscess* OR dentoalveolar AND abscess* OR dental AND abscess* OR dental AND granuloma* OR dentoalveolar AND cyst* OR dentoalveolar AND granuloma* OR radicular AND lesion* OR radicular AND abscess* OR apical AND lesion* OR apical AND abscess* OR acute AND dentoalveolar AND abscess* OR acute AND periapical AND abscess*
**Search #2**	microorganism* OR microbiota OR microbiome* OR anaerobe* OR aerobe* OR pathogen OR pathogens OR germ OR germs OR microbe* OR facultative AND anaerobe* OR microaerophile* OR micro-organism* OR bacteria OR bacterium
**Search #3**	uncultivable OR yet-to-be-cultivated OR unculturable OR uncultured OR uncultivated OR non-culturable OR non-cultivable OR non-cultivated OR non-cultured OR sequenc*
**Search #4**	"16S rRNA gene sequencing" OR "Temperature gradient gel electrophoresis" OR "Single-Strand Conformation Polymorphism" OR "Restriction fragment length polymorphism" OR "Terminal restriction fragment length polymorphism" OR "DNA microarray" OR "Fluorescence in situ hybridization" OR "Quantitative polymerase chain reaction" OR "Sanger Sequencing" OR "first generation of sequencing" OR "next generation sequencing" OR "NGS" OR "Pyrosequencing 454" OR "Illumina's Hi-Seq genome sequencer" OR "Massively parallel signature sequencing" OR "Polony sequencing" OR "SOLiD sequencing" OR "Life Technologies SOLiD" OR "Ion semiconductor" OR "Ion Torrent sequencing" OR "Combinatorial probe anchor synthesis" OR "Nanopore Sequencing" OR "DNA nanoball sequencing" OR "Heliscope single molecule sequencing" OR "Single molecule real time sequencing" OR "DNA nanoball sequencing" OR "Tunnelling currents DNA sequencing" OR "MALDI-TOF MS Mass spectrometry" OR "microfluidic Sanger sequencing" OR "Transmission electron microscopy DNA sequencing" OR "RNA polymerase sequencing" OR "Roche 454" OR "Life Technologies Ion Torrent" OR "Pacific Biosciences" OR "third generation sequencing" OR "polymerase chain reaction" OR "denaturing gradient gel electrophoresis" OR "real time polymerase chain reaction"
**Search #5**	#1 AND #2 AND #3 OR #4

Science direct

Search #1 apical AND lesion* OR granuloma* OR abscess* OR cyst* AND bacteria OR bacterium OR microorganism* OR micro-organism* AND uncultivable OR uncultivated OR unculturable OR uncultured OR non-cultivated OR non-cultivable OR non-cultured OR sequenc* OR non-culturable

Search #2 dentoalveolar lesion* OR dentoalveolar granuloma* OR dentoalveolar abscess* OR dentoalveolar cyst* OR radicular cyst* AND uncultivable OR sequenc* AND bacteria

Search #3 dentoalveolar lesion* OR dentoalveolar granuloma* OR dentoalveolar abscess* OR dentoalveolar cyst* OR radicular cyst* AND unculturable OR uncultivable OR sequenc* AND microorganism*

Search #4 periapical lesion* OR periapical granuloma* OR periapical abscess* OR periapical cyst* OR radicular cyst* AND uncultivable OR unculturable OR sequenc* AND bacteria

Ovid

Search #1 apical AND lesion* OR granuloma* OR abscess* OR cyst* AND bacteria OR bacterium OR microorganism* OR micro-organism* AND uncultivable OR uncultivated OR unculturable OR uncultured OR non-cultivated OR non-cultivable OR non-cultured OR non-culturable OR sequenc*

Search #2 dentoalveolar lesion* OR dentoalveolar granuloma* OR dentoalveolar abscess* OR dentoalveolar cyst* AND uncultivable OR sequenc* AND bacteria

Search #3 dentoalveolar lesion* OR dentoalveolar granuloma* OR dentoalveolar abscess* OR dentoalveolar cyst* AND unculturable OR uncultivable OR sequenc* AND microorganism*

Search #4 periapical lesion* OR periapical granuloma* OR periapical abscess* OR periapical cyst* OR radicular cyst* AND uncultivable OR unculturable OR sequenc* AND bacteria

Filter search Publication date from 1990/01/01 to 2020/05/31; Humans
