# Supplementary material for: Prevalence of unculturable bacteria in the periapical abscess: A systematic review and meta-analysis
Source: PLoS One. 2021 Aug 5;16(8):e0255485. doi: 10.1371/journal.pone.0255485 (PMC8341601; doi:10.1371/journal.pone.0255485)
Supplement: S4 Table — (PDF) [file pone.0255485.s005.pdf]

Studies of acute periapical abscess	Sample and bacterial number *	Unculturable bacteria	Unculturable bacterial clones	Total bacterial clones	Abundance%	Unculturable bacterial frequency	Total sample no.	Frequency %	Sequence techniques
**Flynn et al. (2012) [46] (Abundance/ Frequency)**	9 (5/25)	*Leptotrichia* sp. AM420283	1	391	0.26	1	9	11.11	Sequence
		Leptotrichia [G-1] sp. Oral Taxon 220	1	391	0.26	1	9	11.11	Sequence
		Peptostreptococcaceae [G-4] sp. Oral Taxon 103	2	391	0.51	2	9	22.22	Sequence
		Synergistes[G-3] sp. Oral Taxon 360	1	391	0.26	1	9	11.11	Sequence
		Prevotella sp. Oral Taxon 315	1	391	0.26	1	9	11.11	Sequence
**George et al. (2016) [47] (Frequency)**	18 (4/41)	*Actinomyces* sp clone EP053_ot177_X45	–	–	–	16	18	89	Hybridization
		Peptostreptococcaceae species clone DA014 ot 113 AD07	–	–	–	10	18	55	Hybridization
		Prevotella species clone DO045 ot309 X59	–	–	–	11	18	61	Hybridization
		Acidaminococcaceae species clone DM071_ot135_AC62	–	–	–	17	18	95	Hybridization
**Riggio et al. (2007) [48] (Abundance)**	4 (3/29)	Uncultured Bacteroidetes(phy.)	9	203	4.4	–	–	–	Hybridization
		Uncultured *Prevotella* sp.	2	203	1.0	–	–	–	Hybridization
		Uncultured *Peptostreptococcus*	1	203	0.5	–	–	–	Hybridization
**Rocas et al. (2006) [49] (Frequency)**	77 (1/10)	TM7 clone 1025	–	–	–	2	77	3	Sequence
**Rocas and Siqueira (2009) [50] (Frequency)**	21 (1/3)	Bacteroidetes clone X083	–	–	–	3	21	14	Hybridization
**Rocas and Siqueira (2018) [51] (Frequency)**	55 (2/39)	*Atopobium* sp. HOT-416	–	–	–	3.3	55	6.00	Hybridization
		Orobacterium sp. HOT-102	–	–	–	8.25	55	15.00	Hybridization
**Sakamoto et al. (2006) [52] (Abundance/ Frequency)**	7 (9/29)	Bacteroidales oral clone MCE7_164/MCE3_262/MB4_G15	1	93	1.08	1	7	14	Hybridization
		Prevotella sp. E9_42/*Preqotella* sp. oral clone PUS9.180	2	93	2.15	3	7	43	Hybridization
		Uncultured Eubacterium E1-K13	1	93	1.08	1	7	14	Hybridization
		Lachnospiraceae oral clone 55A-34	1	93	1.08	4	7	57	Hybridization
		Lachnospiraceae oral clone MCE7_60	1	93	1.08	3	7	43	Hybridization

		Peptostreptococcus sp. oral clone CK035	11	93	11.83	2	7	29	Hybridization
		Selenomonas sp. oral clone 55A-7	1	93	1.08	2	7	29	Hybridization
		Bacterium MDA2477/Bacterium MDA2477-like oral clone 51A-9	5	93	5.38	1	7	14	Hybridization
		Pseudomonas sp. LCY11	1	93	1.08	1	7	14	Hybridization
**Sakamoto et al. (2009) [44] (Abundance/ Frequency)**	6 (15/24)	*Treponema* sp. oral taxon IV:18:C9	2	287	0.70	2	6	33.3	Sequence
		Treponema clone 142-10	1	287	0.35	1	6	16.7	Sequence
		Treponema clone 142-21	2	287	0.70	1	6	16.7	Sequence
		Treponema clone 142-82	1	287	0.35	1	6	16.7	Sequence
		Treponema clone 18f-1	2	287	0.70	1	6	16.7	Sequence
		Treponema clone 18f-6	1	287	0.35	1	6	16.7	Sequence
		Treponema clone 18f-7	1	287	0.35	1	6	16.7	Sequence
		Treponema clone 18f-22	1	287	0.35	1	6	16.7	Sequence
		Treponema clone 18f-33	1	287	0.35	1	6	16.7	Sequence
		Treponema clone 18f-35	1	287	0.35	1	6	16.7	Sequence
		Treponema clone 18f-48	1	287	0.35	1	6	16.7	Sequence
		Treponema clone 94A-72	1	287	0.35	1	6	16.7	Sequence
		Treponema clone 94A-89	1	287	0.35	1	6	16.7	Sequence
		Treponema clone 94A-92	1	287	0.35	1	6	16.7	Sequence
		Treponema clone 94A-94	1	287	0.35	1	6	16.7	Sequence
**Siqueira and Rocas (2009) [54] (Frequency)**	42 (11/55)	Bacteroidetes clone X083	–	–	–	36	42	15.12	Hybridization
		Lachnospiraceae 55A-34	–	–	–	12	42	5.04	Hybridization
		Treponema II:10: D12	–	–	–	12	42	5.04	Hybridization
		Dialister clone 55A-29	–	–	–	8	42	3.36	Hybridization
		Dialister clone 9N-7	–	–	–	5	42	2.1	Hybridization
		Lachnospiraceae clone MCE7_60	–	–	–	5	42	2.1	Hybridization
		Synergistes clone BH007	–	–	–	5	42	2.1	Hybridization
		Treponema 6:H:D15A-4	–	–	–	5	42	2.1	Hybridization
		Megasphaera clone CS025	–	–	–	3	42	1.26	Hybridization
		Olsenella genomospecies C1	_	_	_	3	42	1.26	Hybridization
		Treponema I:G:T21 /I:W:T040	_	_	_	3	42	1.26	Hybridization

**Yang et al. (2010) [55] (Abundance/ Frequency)**	11 (1/17)	*Bacteroidales genomosp*. P4 oral clone MB2_G17	8	424	1.9	1	11	9.1	Sequence
**Zhang et al. (2020) [56] (Abundance/ Frequency)**	9 (6/125)	*Acinetobacter* sp. Oral taxon 408	5.1	681	0.75	3	9	33	Sequence
		Peptostreptococcaceae [XI][G-7] sp. oral taxon 081	3.8	681	0.56	1	9	11	Sequence
		Uncultured bacterium clone JN379053	_	_	_	_	_	_	Sequence
		Uncultured bacterium clone JN379054	_	_	_	_	_	_	Sequence
		Uncultured bacterium clone JN379056	_	_	_	_	_	_	Sequence
		Uncultured bacterium clone JN379057	_	_	_	_	_	_	Sequence
**Total**	259 (58/397)								
**Studies of periapical abscess**									
**Vengerfeldt et al. (2014) [59] (Frequency)**	4 (1/54)	TG5 group	_	_	_	1	4	25	Sequence
**Jacinto et al. (2007) [60] (Abundance /Frequency)**	5 (3/33)	Uncultured *Staphylococcus* sp. clone pGA 2	10	480	2.08	2	5	40	Hybridization
		Uncultured rape rhizosphere bacterium wr0200	13	480	2.71	1	5	20	Hybridization
		Uncultured bacterium clone aab38e07	7	480	1.46	1	5	20	Hybridization
**Total**	9 (4/87)								

*The samples numbers: The number of samples in a study (the number of the uncultured bacteria/the total number of bacteria identified).
