# Supplementary figures and images for: Prevalence of unculturable bacteria in the periapical abscess: A systematic review and meta-analysis
Source: PLoS One. 2021 Aug 5;16(8):e0255485. doi: 10.1371/journal.pone.0255485 (PMC8341601; doi:10.1371/journal.pone.0255485)

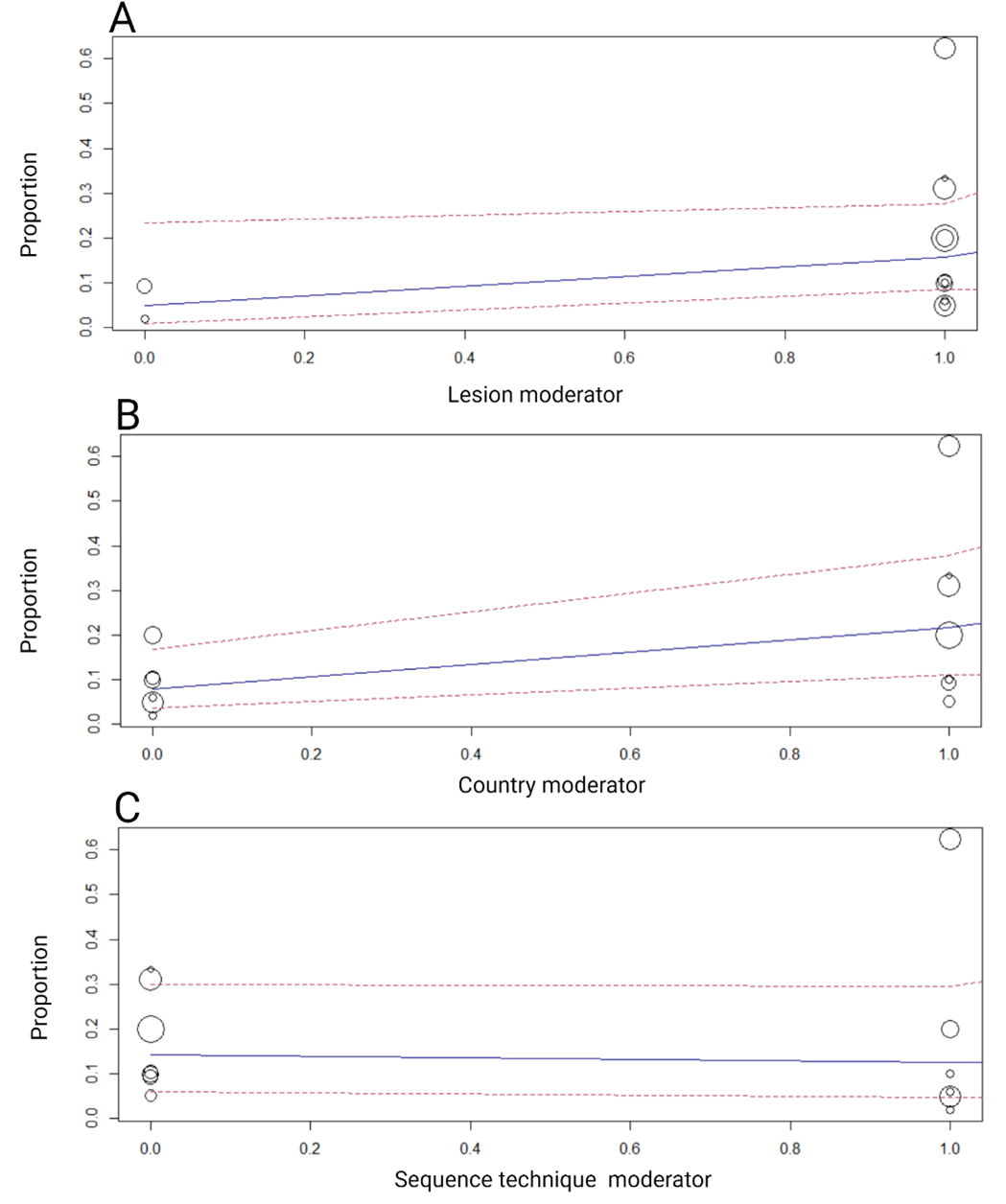

Supplement: S1 Fig — (A) Lesion moderator. (B) Country moderator. (C) Sequence technique moderator. (TIF) [file pone.0255485.s006.tif]

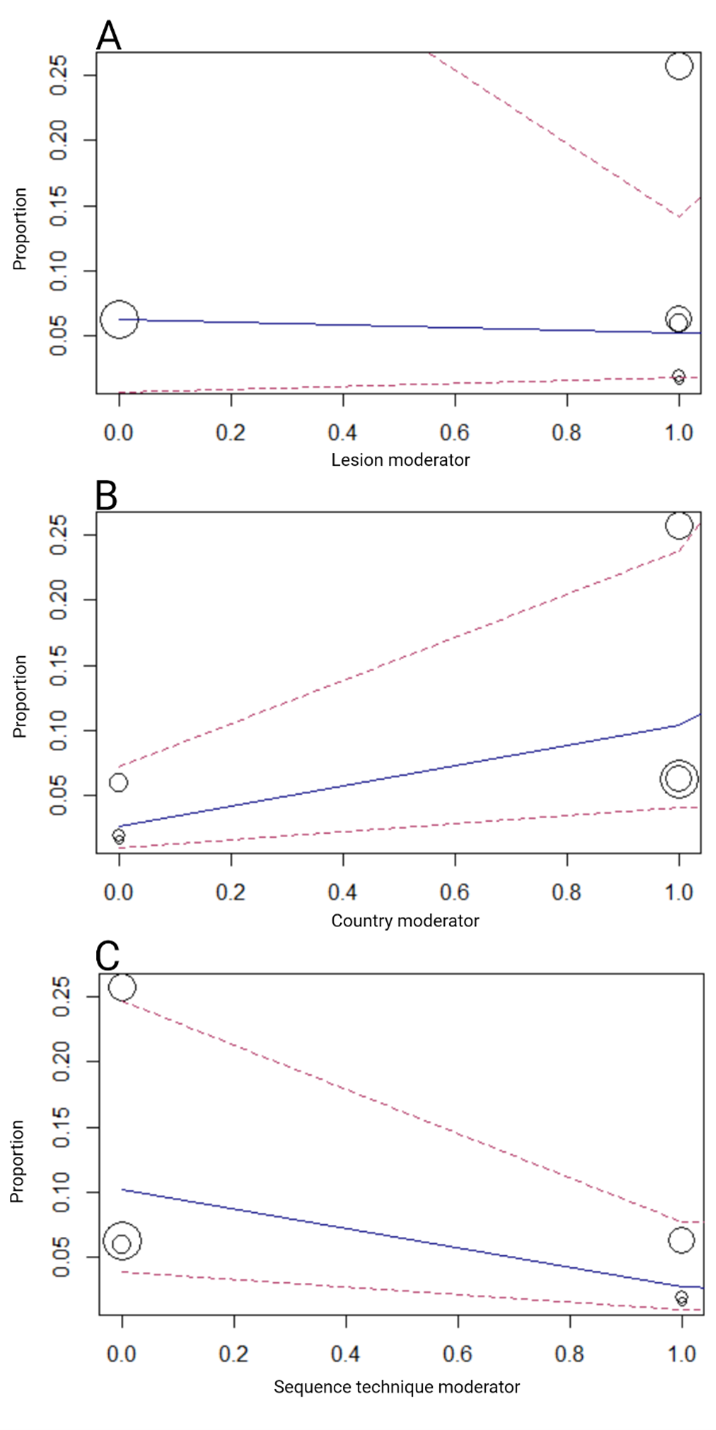

Supplement: S2 Fig — (A) Lesion moderator. (B) Country moderator. (C) Sequence technique moderator. (TIF) [file pone.0255485.s007.tif]

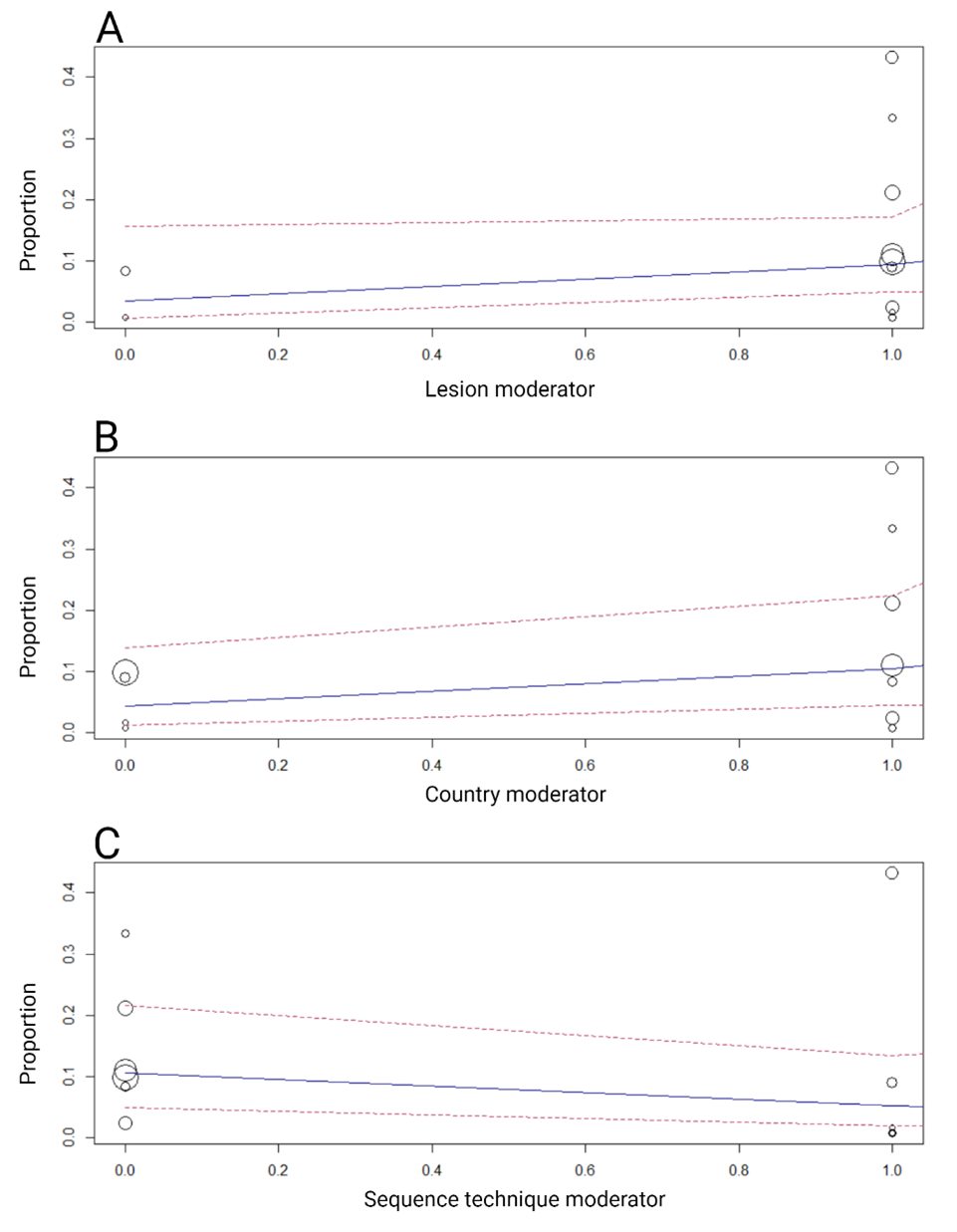

Supplement: S3 Fig — (A) Lesion moderator. (B) Country moderator. (C) Sequence technique moderator. (TIF) [file pone.0255485.s008.tif]

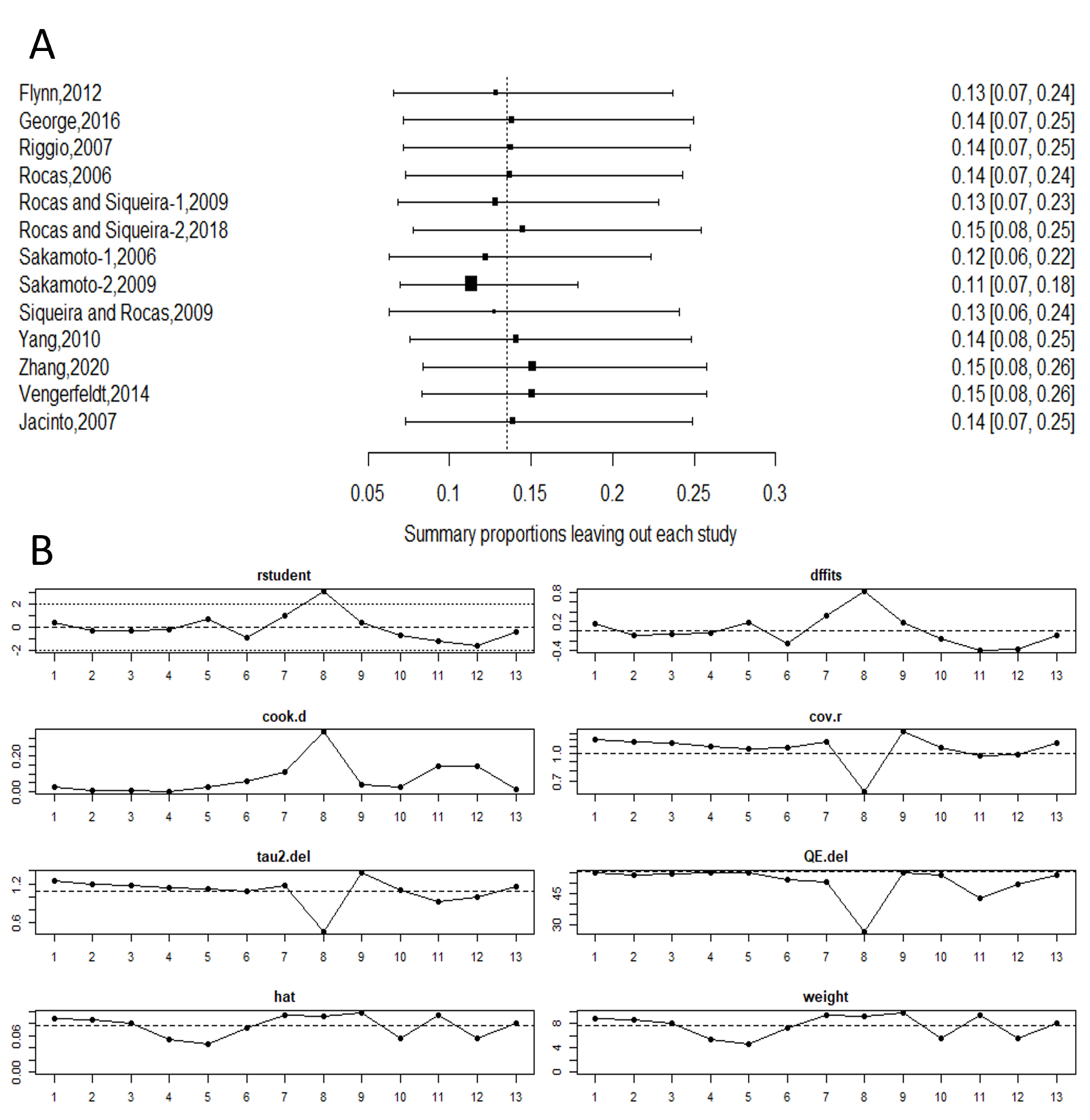

Supplement: S4 Fig — (A) Forest plot depicting summary proportion after leaving out each study. (B) Influential analysis plot of diversity of unculturable bacteria in thirteen studies of periapical abscess. (TIF) [file pone.0255485.s009.tif]

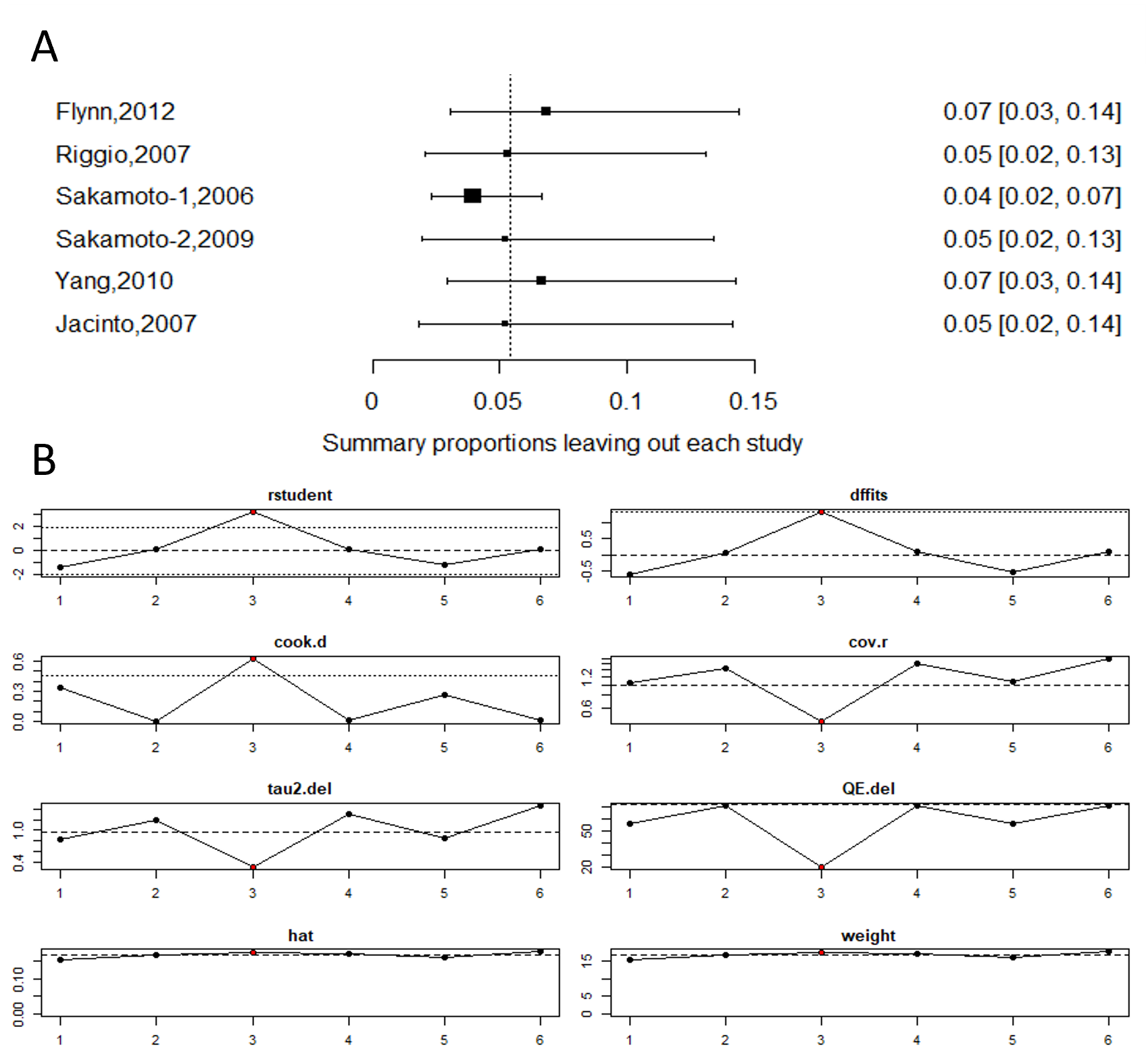

Supplement: S5 Fig — (A) Forest plot depicting summary proportion after leaving out each study. (B) Influential analysis plot of abundance of unculturable bacteria in six studies of periapical abscess. (TIF) [file pone.0255485.s010.tif]

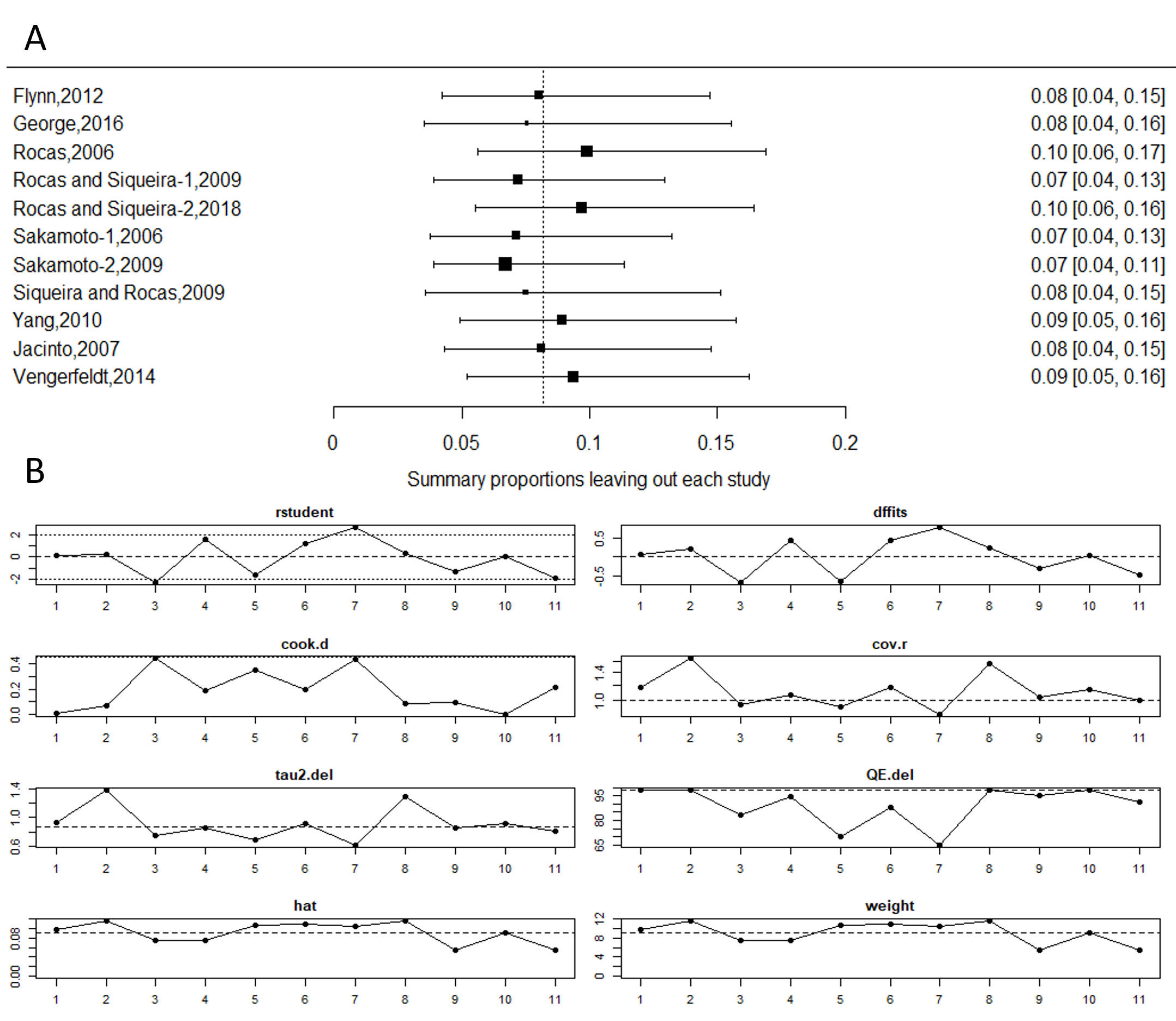

Supplement: S6 Fig — (A) Forest plot depicting summary proportion after leaving out each study. (B) Influential analysis plot of frequency of unculturable bacteria in eleven studies of periapical abscess. (TIF) [file pone.0255485.s011.tif]

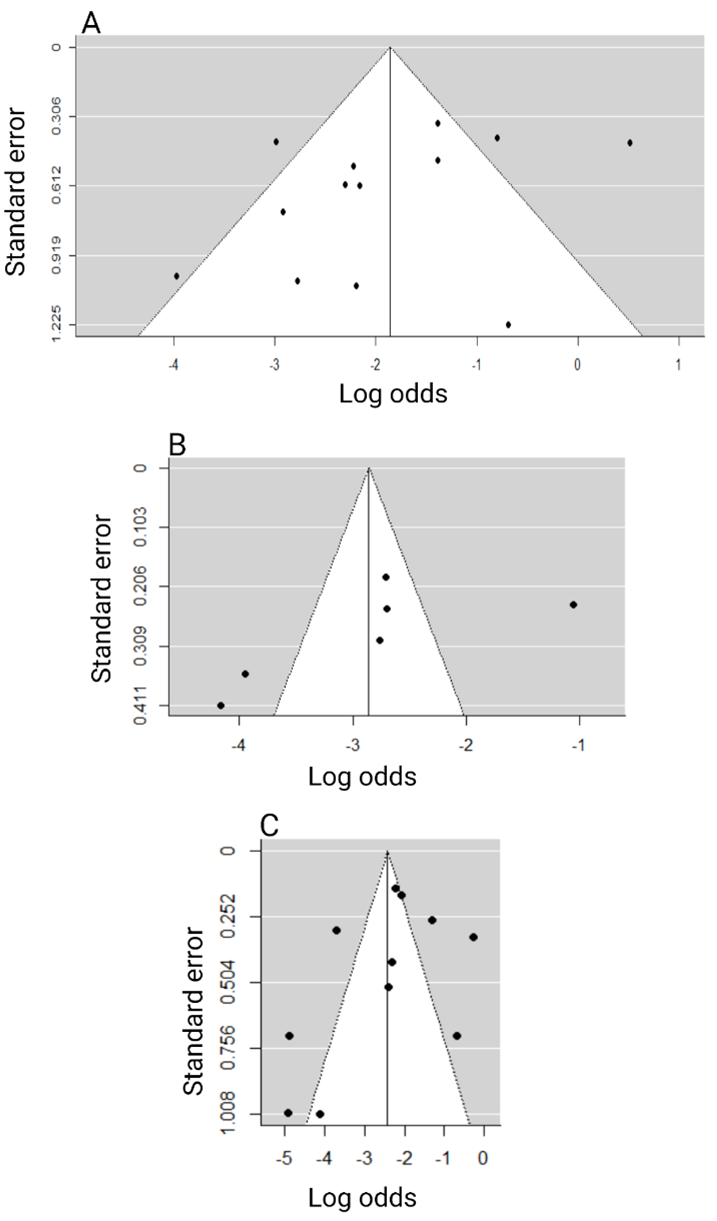

Supplement: S7 Fig — (A) Diversity. (B) Abundance. (C) Frequency. (TIF) [file pone.0255485.s012.tif]

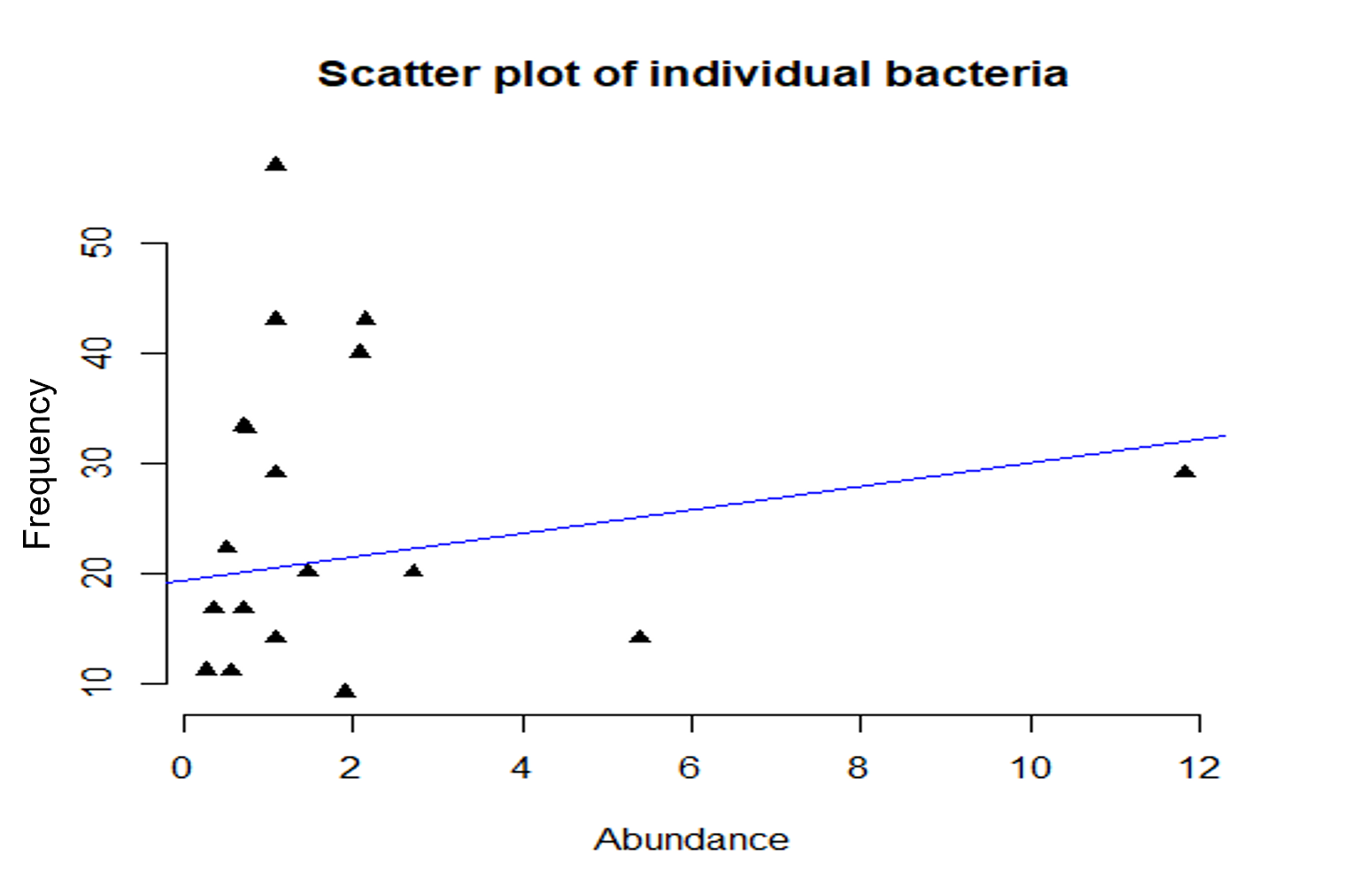

Supplement: S8 Fig — (TIF) [file pone.0255485.s013.tif]
