# Supplementary material for: Prevalence of unculturable bacteria in the periapical abscess: A systematic review and meta-analysis
Source: PLoS One. 2021 Aug 5;16(8):e0255485. doi: 10.1371/journal.pone.0255485 (PMC8341601; doi:10.1371/journal.pone.0255485)
Supplement: Attachment — Submitted filename: Reviewer 4.docx [file pone.0255485.s014.docx]

The authors are commended for their efforts to carry out this systematic review on the prevalence of uncultivable bacteria in dentoalveolar abscess. Although this article addresses an important issue in endodontic microbiology, there are major concerns regarding the study desing and interpretation of the data (please see comments below). Moreover, the conclusions are only partially supported by the data.

Title:

This systematic review focused on apical abscesses. As no data on “related periapical lesions” was provided, I would suggest to remove this part of the title.

Introduction:

Lines 52-61: The authors used Nair (1997) as a reference for the classification of apical diseases. However, as the diagnosis in Endodontics is based on clinical signs/ symptoms and not on histopathological findings, an updated classification of apical diseases would be more appropriate (https://www.aae.org/specialty/clinical-resources/glossary-endodontic-terms/).

Line 69: The “foregoing organisms” were not mentioned the text. Please revise this sentence.

Line 88: Granuloma and cysts are not sequelae of apical abscess. Please revise this sentence.

Lines 90-91: The secondary aim of this study was to review the possible role of non-cultivable microorganisms in the pathogenesis of apical diseases. However, the presence or abundance of bacterial DNA does not necessarily represent the bacterial activity in the disease process, as the DNA of dead bacteria can be detected by molecular methods. In addition, it has been found that bacteria with low abundance can be active in oral infections. Therefore, the secondary objective cannot be achieved due to the limitations of the studies included in this review.

Materials and Methods:

Lines 112-113: If the AAE classification of apical periodontitis (i.e., asymptomatic apical periodontitis) had been used instead of the classification based on histological findings (i.e., periapical  granuloma and cyst), important information could have been extracted from the 60 articles that were excluded.

Line 209: Please explain which periapical lesions are associated with DAA.

Outcome measures:

Lines 395-405: Due to the high diversity of the microbiome among individuals, most bacterial taxa were found only once. Therefore, the frequency and abundance analysis at the species level may not provide representative data.

Lines 406-409: The statement that hybridization techniques are the most reliable molecular methods for detecting noncultivable bacteria should be analyzed with caution. First, it is important to note that only 5 studies were included in this analysis. Second, this finding is in contrast to the literature on microbiological methods, showing that sequencing is a reliable method for studying the oral microbiome. These issues should be further discussed.

Conclusion:

Line 479: Bacterial abundance may not be correlated with their activity (please see the comments above: Lines 90-91). Please revise this sentence.
